# Genetic and Antigenic Characteristics of Highly Pathogenic Avian Influenza A(H5N8) Viruses Circulating in Domestic Poultry in Egypt, 2017–2021

**DOI:** 10.3390/microorganisms10030595

**Published:** 2022-03-09

**Authors:** Ahmed Kandeil, Yassmin Moatasim, Ahmed El Taweel, Mohamed El Sayes, Adam Rubrum, Trushar Jeevan, Pamela P. McKenzie, Richard J. Webby, Mohamed A. Ali, Ghazi Kayali, Rabeh El-Shesheny

**Affiliations:** 1 Center of Scientific Excellence for Influenza Viruses, National Research Centre, Giza 12622, Egypt; ahmed.kandeil@stjude.org (A.K.); yasmin.moatasim@human-link.org (Y.M.); ahmed.nageh@human-link.org (A.E.T.); mohameddiaaelsayes@outlook.com (M.E.S.); mohamedahmedali2004@yahoo.com (M.A.A.); 2 Department of Infectious Diseases, St. Jude Children’s Research Hospital, Memphis, TN 38105, USA; adam.rubrum@stjude.org (A.R.); trushar.jeevan@stjude.org (T.J.); pamela.mckenzie@stjude.org (P.P.M.); 3 Human Link, Dubai 971, United Arab Emirates

**Keywords:** Egypt, highly pathogenic avian influenza, H5N8, phylogenetic analysis, reassortment

## Abstract

In Egypt, the endemicity of avian influenza viruses is a serious concern. Since 2016, several outbreaks of H5N8 have been recorded among domestic poultry in various areas of the country. Active surveillance of domestic poultry across several governorates in Egypt from 2017 to 2021 detected at least six genotypes of Highly Pathogenic Avian Influenza (HPAI) H5N8 viruses with evidence of partial or complete annual replacement of dominant strains. Although all Egyptian H5N8 viruses had clade 2.3.4.4b hemagglutinin (HA) genes, the remaining viral gene segments were from multiple geographic origins, indicating that the H5N8 isolates resulted from multiple introductions. Mutations in the viral proteins associated with pathogenicity and antiviral drug resistance were detected. Some mutations in the HA resulted in antigenic drift. Heterogeneity in circulating H5N8 HPAI threatens poultry production and public health.

## 1. Introduction

Highly Pathogenic Avian Influenza (HPAI) H5 viruses were first detected in chickens in Scotland in 1959 and sporadically thereafter, with limited spread at each instance [1]. Then in 1996, the HPAI A/goose/Guangdong/1/96 (H5N1) virus was detected. It caused death in infected domestic and wild birds and acquired the ability to cross the species barrier and infect humans [2,3]. Descendants of these viruses spread globally, continued circulating, and have evolved into multiple genetically distinct clades (0–9) and subclades. In 2008, the HP H5 clade 2.3.4 was first identified in China and has since evolved into several clades including 2.3.4.4, which was detected in 2013 in China. The HPAI viruses bearing an HA of clade 2.3.4.4 have acquired several types of neuraminidases (NAs) including N1, N2, N5, N6, and N8 by reassortment events with other Low Pathogenic Avian Influenza Viruses (LPAIVs) circulating in wild birds and have further diversified into eight genetically distinct groups (a to h). The 2.3.4.4b viruses were initially detected in China and South Korea during 2013–2014. However, in the second half of 2016, this group was detected in wild birds in several countries in Asia, Europe, and Africa [4,5]. In contrast to the global spreading of groups a and b, other genetic groups (c to h) have a limited geographical distribution.

The 2.3.4.4b H5N8 viruses further evolved after their distribution through active reassortment, with locally circulating LPAIVs forming different geographically defined genotypes. Compared to the parental H5N1 HPAIV, some strains of H5N8 viruses showed lower virulence, reduced transmission, and a longer mean time of death [6,7].

In Egypt, the endemicity of AIVs is a grave concern, with approximately 40,000 poultry farms being at risk of viral infection, given their high prevalence as backyard farms. Egypt is located at the intersection of two migratory birds flyways, the Black Sea–Mediterranean Flyway and the East Africa–West Asia Flyway [8]. Egypt is therefore considered a hot spot for evolution of the influenza virus. The 2.3.4.4b H5N8 virus was first detected in Egypt in migratory birds (common coot and green-winged teal) in late 2016 [9,10]. Since then, several cases of H5N8 have been recorded among domestic poultry in live bird markets, backyard flocks, and commercial farms in several governorates in Egypt. Although all Egyptian H5N8 isolates belong to the clade 2.3.4.4, several independent introductions of the virus have been detected [11,12].

In this study, we identify the genetic and antigenic characteristics of HPAI H5N8 viruses collected during active surveillance activities in Egypt between 2017 and 2021.

## 2. Materials and Methods

### 2.1. Sample Collection and Virus Isolation

A total of 13,104 swabs samples were collected from various species of poultry during 2017–2021 as part of an ongoing long-term active surveillance for avian influenza viruses as previously described [13,14]. Approximately, 50.3% of samples were from commercial poultry farms, 29.6% from live bird markets, and 20.1% from backyard flocks. All collected samples were individually injected into the allantoic cavity of 10-day-old specific pathogen-free embryonated hens’ eggs, incubated for 48 h post-injection at 37 °C, and then chilled at 4 °C for 4 h. Allantoic fluid was then collected and analyzed by the hemagglutination assay (HA) using 0.5% chicken red blood cells (RBCs). Positive HA samples then underwent molecular characterization by RNA extraction using the QIAamp Viral RNA Mini kit (Qiagen, Hilden, Germany) according to the manufacturer’s protocol, followed by typing and subtyping using RT-PCR [15]. We successfully characterized 161 H5N8 isolates (1.22% isolation rate). All isolates were subjected to full genome sequencing. A total of 96 Egyptian HPAI H5N8 viruses from different species of domestic poultry (72 isolates from chickens, 19 from ducks, 1 from a goose, and 4 from pigeons) from January 2017 to February 2021 (29 isolates in 2017, 33 in 2018, 29 in 2019, 1 in 2020, and 4 in 2021) were obtained. The samples were collected from seven governorates in Egypt: Sharqeia (1), Sohag (2), Kalyobiya (16), Monofiya (2), Fayoum (15), Menia (43), and Assiut (17). Details of the isolation area, health status of the host, date of isolation, and site of sampling (commercial farm, market, house, or abattoir) for samples that yielded isolates are given in Table S1.

### 2.2. Sequencing and Sequence Analysis

Extracted RNA from H5N8 isolates were subjected to reverse transcription to synthesize the first cDNA strand using the Superscript III system (Life Technologies, Carlsbad, CA, USA) with Uni 12 primers. Then, Phusion high-fidelity DNA polymerase (New England Biolabs, Ipswich, MA, USA) and universal conserved Uni12/13 primers for influenza A viruses were used for amplification of the full eight gene segments of Egyptian H5N8 viruses, and PCR products were purified using GFX PCR DNA and Gel Band Purification Kit (Cytiva, Amersham, UK). The staff of the Hartwell Center at St. Jude Children’s Research Hospital prepared the DNA libraries using Nextera XT DNA Library Prep Kit (Illumina, San Diego, CA, USA), which were then pooled and sequenced via 150 bp paired end reads by using an Illumina MiSeq personal genome system (Illumina, San Diego, CA, USA). The eight full segments of each H5N8 virus were assembled using CLC Genomics Workbench, version 20 (CLC Bio, Qiagen, Hilden, Germany).

Sequences were deposited in GenBank under the accession numbers listed in Table S2.

Publicly available sequences of the H5 virus clade 2.3.4.4 (a to h) full genome of the reference strains and all H5N8 sequences from Egypt were downloaded from the Global Initiative on Sharing All Influenza Data (GISAID) (http://platform.gisaid.org/epi3/, accessed on 1 August 2021. BLASTN homology analysis of nucleic acids was performed on the GISAID website, and globally related sequences were downloaded. Nucleotide sequences of each segment were collected and underwent multiple alignments using the ClustalW multiple alignment accessory application in BioEdit software version 7.2.5 (Bioedit Company, Manchester, UK).

Aligned sequences were then used to build a phylogenetic tree by using the MEGA X software (Microsoft Windows, Redmond, WA, USA) through a neighbor-joining tool with Kimura’s two-parameter distance model and 1000 replicates in bootstrap. Aligned amino acid sequences of each protein were used to determine the genetic signature markers correlated with virulence, host adaptation, and antigenic sites. Aligned nucleotide and protein ungapped sequences were tested for similarity using MegAlign of the DNASTAR Lasergene 15 software (Madison, WI, USA).

### 2.3. Antigenic Analysis

Selected H5N8 isolates were analyzed by hemagglutination inhibition (HI) assay against reference postinfection ferret antisera raised against F.2015-48-A/Sichuan/26221/2014 (H5N6), A/duck/England/36254/2014 (H5N8), A/chicken/Japanese Kumamoto/1-7/2014 (H5N8), A/gyrfalcon/Washington/410886/2014 (H5N8), A/chicken/Vietnam/NCVD-15A59/2015 (H5N6), A/Hubei/29578/2016 (H5N6), A/Fujian-Sanyuan/21099/2017 (H5N6), and A/snow goose/Missouri/CC15-84A/2015 (H5N2) of clade 2.3.4.4 viruses [16]. For HI assays, 0.5% chicken red blood cells were used. The HAI results were then analyzed by antigenic cartography.

## 3. Results

During our active surveillance of domestic poultry in Egypt, 96 HPAI H5N8 viruses were isolated from dead (43.75%) and healthy (56.25%) birds between 2017 and 2021. The details of the isolation area, the health status of the host, the date of isolation, and the GenBank accession numbers for subsequent sequences are provided in Tables S1 and S2.

### 3.1. Phylogenetic Analysis and Sequence Similarity

**Figure 1:**
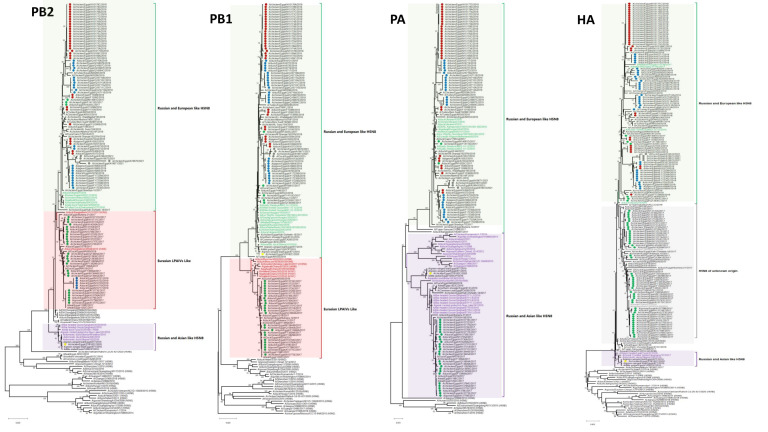

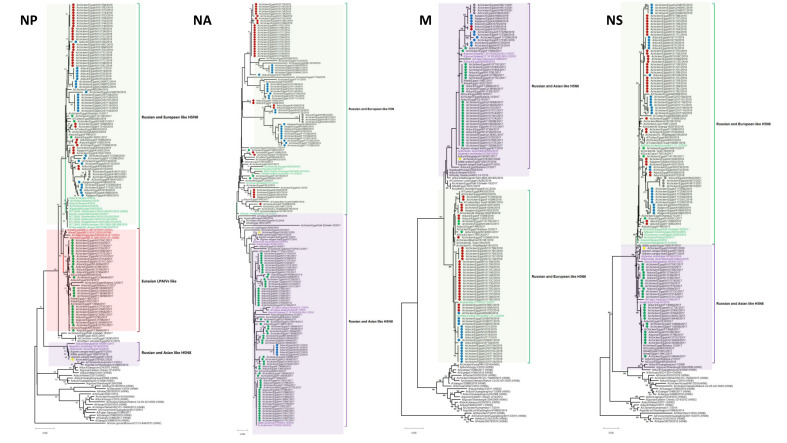
Phylogenetic tree of the nucleotide sequences of eight segments of H5N8 viruses. Isolates sequenced specifically for this study are indicated by circles with green, red, blue, yellow, and gray colors for 2017, 2018, 2019, 2020, and 2021, respectively. International related sequences of Egyptian H5N8 viruses were included and distinguished by text color. Phylogenetic analysis was performed using the neighbor-joining algorithm with the Kimura two-parameter model. The reliability of phylogenetic inference at each branch node was estimated by the bootstrap method with 1000 replications. Evolutionary analyses were conducted in MEGA X.

No reassortment event was characterized through our extensive analysis of 96 Egyptian HPAI H5N8 viruses of the current study and endemic AI H5N1 and H9N2 viruses in Egypt. The similarity of nucleotide and deduced amino acid sequences for each segment among different characterized H5N8 viruses of the current study were determined using MegAlign. The PB2 similarities among the Egyptian H5N8 viruses ranged from 96.9% to 100% and 97.6% to 100% at the nucleotide and amino acid levels, respectively. The PB2s clustered into three phylogenetically distinct groups (Figure 1): the Russian- and European-like H5N8 group included Egyptian viruses from 2017, 2018, 2019, and 2021, The Russian- and Asian-like H5N8 group included an Egyptian strain from 2020, and the Eurasian LPAIV group included Egyptian H5N8 viruses from 2017 and 2019. The PB1 nucleotide and deduced amino acid sequence similarities of the Egyptian viruses ranged from 94.8% to 100% and 97% to 100%, respectively. Phylogenetic analysis of the PB1 gene revealed that the 96 Egyptian strains were clustered into two main groups. The first group included most of the viruses isolated in 2017, which were more related to Eurasian LPAIVs than to the H5N8 progenitors. The second group of Egyptian viruses isolated from 2017 to 2021 clustered with Russian and European H5N8 viruses. Analysis of the PA genes showed that the nucleotide and deduced amino acid sequence similarities among Egyptian strains ranged from 92.9% to 100% and 97.8 to 100%, respectively. The PAs of the Egyptian isolates clustered into two main groups: H5N8 isolates from Russian and Asian (2017) and Russian and European (2017–2021) strains. The HA nucleotide and deduced amino acid sequence similarities among Egyptian strains ranged from 93.4% to 100% and 98.8% to 100%, respectively. The HAs were clustered in clade 2.3.4.4b with the reference strain A/Fujian-Sanyuan/21099/2017 (H5N6) (Figure 1). The HA and NP of Egyptian H5N8 viruses were classified into three subgroups (I, II, and III). This clustering was associated with the year of isolation and not with the geographical location or host of isolation. Subgroup I included some Egyptian isolates from 2017 and other H5N8 viruses from different European countries. Subgroup II included isolates from 2017 to 2019 and from 2021, which were similar to Russian and European H5N8 viruses characterized in 2016. However, subgroup III included only the A/chicken/Egypt/S18182C/2020 (H5N8) virus that was closely related to the A/green-winged teal/Egypt/871/2016 (H5N8) virus derived from Russian- and Asian-like H5N8 viruses. The NP nucleotide and amino acid sequence similarities ranged from 97.5% to 100% and 98.8% to 100%, respectively. Phylogenetically, the Egyptian NA genes clustered into two groups (Russian- and European-like H5N8 (2017, 2019) and Russian- and Asian-like H5N8 (2017–2021)) (Figure 1) within the larger 2.3.4.4b clade with nucleotide and amino acid sequence similarity ranging from 96.9% to 100% and from 95.8% to 100%, respectively. The M segment evolved into two subgroups, with a nucleotide sequence similarity ranging from 97% to 100%. The amino acid sequence similarity ranged from 96.4% to 100% for M1 and from 96.9% to 100% for M2. The NS segments of Egyptian H5N8 viruses were clustered in two main groups: the first group included most of the isolates from 2017 and an isolate from 2020, and the second group included the 2017–2021 isolates (Figure 1). The NS gene nucleotide sequence similarity ranged from 96.3% to 100%, and the amino acid sequence similarity ranged from 97.2% to 100% for NS1 and 97.5% to 100% for NS2.

### 3.2. Genetic Characterization

#### 3.2.1. Internal Segments

All Egyptian H5N8 viruses maintained the avian virus signatures at positions 627 and 701 of PB2 (K and N, respectively) (Table 1). However, all isolates displayed some mammalian adaptation and virulence features such as PB2 (504V), PB1 (13P), PA (672L), NP (398Q), M2 (64S), and NS1 (42S) [17,18,19,20,21] (Table 2). Moreover, most isolates had the PA (127V) mammalian virulence mutation. The NS1 PDZ motif GSEV (227–230) was present in approximately 22.9% of the Egyptian H5N8 viruses. The isolates from 2021 displayed two mammalian virulence mutations that were not present in published Egyptian H5N8 sequences: NS1 189N in four isolates and NS2 31I in two isolates. The M2 sequence in all Egyptian H5N8 isolates lacked the amantadine-resistance mutation (S31N), and only five sequences displayed the (V27A) resistance mutation [22].

#### 3.2.2. HA

The HA genes of the H5N8 Egyptian viruses maintained Q222 and G224 (H5 numbering), which is suggestive of preferential binding to avian-like receptors over human-like receptors. Most viruses displayed the multiple basic amino acid motif “PLREKRRKR/GLF” in the cleavage site, whereas two H5N8 isolates, A/duck/Egypt/F15089/2018 and A/duck/Egypt/F15092/2018, had “PPRGKRRKR/GLF”. All HPAIV H5N8 viruses had five potential N-linked glycosylation sites at positions 10, 23, 156, 483, and 542. Several mutations were recorded at antigenic sites A and B. Fifteen percent of HA sequences displayed a T140A mutation in antigenic site A, whereas three sequences displayed a T140V mutation. One A/duck/Egypt/Q16716A/2019(H5N8) virus had an A154D substitution in the antigenic site B. Interestingly, seven Egyptian H5N8 viruses from H5N1 and H9N2 vaccinated farms in the same governorate had the N183S substitution in the antigenic site B. A/pigeon/Egypt/A16800/2019(H5N8) had a substitution (S181P) in site B.

#### 3.2.3. NA

All the Egyptian H5N8 viruses had full-length NAs with no evidence of deletions, and the N-linked glycosylation sites at 54, 67, 84, and 144 were conserved. However, the glycosylation site at 293 (NWT) was lost in 10.2% of all Egyptian sequences. This loss was present in the four 2021 sequences [10]. A new potential glycosylation site (42 NGT) was found in 40.4% of the isolates. All Egyptian N8 sequences had the 312V mutation responsible for oseltamivir resistance [46]. No other oseltamivir or zanamivir resistance markers [46] were found.

### 3.3. Genotyping of the Egyptian H5N8 Viruses

**Figure 2:**
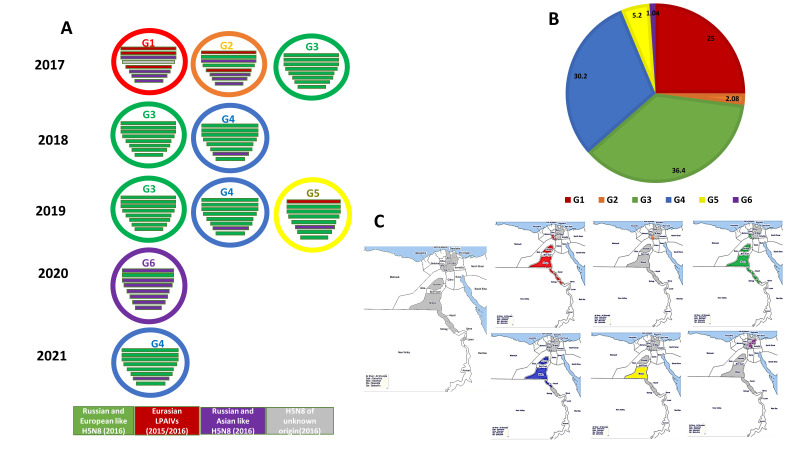
(**A**) Genetic constellation of six Egyptian HPAI H5N8 genotypes identified between 2017 and 2021 based on the genetic analysis of full-length genome sequences. (**B**) Pie chart showing the prevalence rate of different HPAI H5N8 genotypes. (**C**) Geographical distribution of different H5N8 genotypes.

At least six different genotypes (G1 to G6) of the 2.3.4.4b H5N8 viruses were represented in the viruses isolated and characterized from Egypt (Figure 2). The six genotypes were identified in different types of poultry production sectors through our active surveillance study from 2017 to 2021. Of 96 Egyptian H5N8 viruses characterized, 24 isolates (25%) were classified as Genotype 1 (G1) and were detected only in 2017 (prior to June) in six governorates, including two Nile Delta governorates (Monofiya [one isolate from vaccinated chicken in a farm], and Kalyobiya [two isolates from vaccinated chickens in a farm]); Fayoum in Middle Egypt (three isolates from farms); and three Southern Egypt governorates (Assiut [four isolates from backyard flocks (two isolates) and farms (two isolates)]), Menia (ten isolates from backyard flocks and two isolates from markets), and Sohag (two isolates from farms). The genome constellation of the G1 viruses detected in Egypt was closely related to Egyptian H5N8 viruses detected in wild birds in Egypt, A/teal/Egypt/1198C/2017 (H5N8) and A/teal/Egypt/1202C/2017 (H5N8), and distinct from that of the reassortant H5N8 viruses detected in Eurasian countries during 2016 and early 2017. The PB2, PB1, and NP gene segments of the G1 Egyptian H5N8 viruses were closely related to Eurasian LPAIVs identified in wild birds during 2015 and 2016. The PA, NA, M, and NS genes were likely to be derived from Russian- and Asian-like HPAI H5N8 viruses identified in 2016, such as A/duck/India/10CA01/2016 (H5N8) and A/domestic duck/Siberia/49 feather/2016 (H5N8). By June 2017, G1 of Egyptian H5N8 viruses were not detected during our surveillance study. Only two H5N8 viruses, A/chicken/Egypt/Q13936B/2017 (H5N8) and A/chicken/Egypt/Q13936C/2017 (H5N8), identified only in 2017 in vaccinated chickens in farms in the Kalyobiya governorate belonged to Genotype 2 (G2). The genome constellation of G2 H5N8 viruses showed that the genome of H5N8 viruses was similar to that of the G1 viruses except the PB1 and HA segments had different origins (Russian- and European-like H5N8 viruses). Both G1 and G2 were not detected in poultry after 2017.

The dominant genotype of the H5N8 viruses was G3 (36.4%), which was initially detected in three different farms in three governorates (Monofiya, Fayoum, and Menia) in 2017, with their detections increasing gradually in 2018. The G3 viruses were detected in Assiut (live bird market (*n* = 3)), Fayoum (live bird market (*n* = 4), farm (*n* = 2)), and Menia (backyard flocks (*n* = 24)). In 2019, a single G3 virus was detected in dead vaccinated chicken in a farm in the Kalyobiya governorate. In contrast to G1 and G2 viruses that were identified in healthy poultry, 71.4% of G3 viruses were collected from dead chickens, suggesting a correlation with increased pathogenicity. The G3 viruses were completely distinct from the G1 viruses, and the whole genome was closely related to the Russian and European H5N8 viruses of 2016.

The second most abundant genotype of H5N8 viruses detected was G4 (30.2%), which was detected in 2018 (2 isolates), 2019 (23 isolates), and 2021 (4 isolates). G4 was detected in four governorates including Kalyobiya (11 isolates from vaccinated chickens and ducks in several farms), Fayoum in Middle Egypt (six isolates from vaccinated chicken in farms), and two Southern governorates (Assiut (10 isolates from live bird markets), and Menia (2 isolates from live bird markets)). Similar to the G3 viruses, 58.6% of the G4 viruses were collected from dead chickens and ducks. The only difference between the G4 and G3 was the origin of the M segment.

The G5 Egyptian H5N8 viruses made up 5.2% of the characterized viruses and were detected only in healthy ducks at a live bird market in the Menia governorate in 2019. The G5 viruses had the same genetic origin as G3 in PB1, PA, HA, NP, M, and NS, whereas the PB2 and NA viral segments had the same origin as G1 and G2.

One H5N8 isolate detected in 2020 was similar to the viruses responsible for the first incursion of H5N8 through wild birds in Egypt, its gene segments were found to be closely related to A/green-winged teal/Egypt/871/2016 (H5N8).

Notably, genotypes 1, 3, and 4 viruses were detected in several species of poultry, indicating interspecies transmission. Genotypes 2, 5, and 6 were only detected in chickens and/or ducks.

### 3.4. Antigenic Analysis

**Figure 3:**
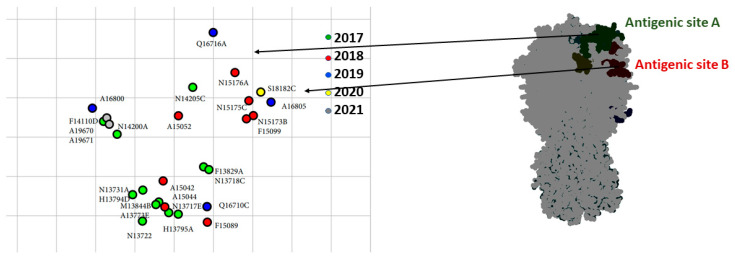
Antigenic mapping based on the cross-reactivity of Egyptian H5 clade 2.3.4.4b viruses, with reference sera and 3D structure model of HA displayed in antigenic sites. Some strains drifted in cross-reactivity with reference sera due to mutations in antigenic sites A and B.

Selected viruses were tested antigenically against reference strains of clade 2.3.4.4 H5 viruses (Table S3). These data revealed that some detected viruses had undergone antigenic drift due to the accumulation of mutations in antigenic sites A (T140A, T127I) and B (N154D, S181P). The cartograph of the antigenic data is shown in Figure 3. Most of the Egyptian isolates had an antigenic profile similar to that of A/chicken/Japanese Kumamoto/1-7/2014 (H5N8) (Table S3). Over 90% of the Egyptian viruses reacted well to sera raised against A/gyrfalcon/Washington/41088-6/2014 (H5N8), A/duck/England/36254/2014 (H5N8), and A/chicken/Japanese Kumamoto/1-7/2014 (H5N8), with titers of 40–320, and to A/snow goose/Missouri/CC15-84A/2015 (H5N2), with titers of 10–160.

## 4. Discussion

Of all A/goose/Guangdong/1/96 clades, those of 2.3.4 showed a higher propensity to reassort, forming several genotypes of H5NX viruses. At the end of 2016, two different reassortants of 2.3.4.4b H5N8 viruses were detected in wild birds (common coot and green-winged teal) in Egypt [9,10]. Soon after, several HPAI H5N8 viruses were reported in domestic poultry in Egypt as a result of multiple introductions [11,13,47,48]. The early evidence of multiple introductions and reassortment and the endemicity of H5N1 and H9N2 viruses in Egypt led us to expect a complicated epidemiological situation. However, the prevalence of H5N1 has gradually declined and is being replaced by H5N8 viruses. Novel H5N2 reassortants between Egyptian HPAI H5N8 and H9N2 viruses have, however, been detected in limited cases in domestic poultry [49,50].

In the current study, we determined the genetic and antigenic characteristics of HPAI H5N8 viruses detected in domestic poultry in Egypt from the first wave of introduction in domestic poultry until 2021. The viruses were collected from farms, backyards, and markets during our long-term active surveillance of AIVs from 2017 to 2021. The HA of all Egyptian H5N8 strains belonged to clade 2.3.4.4b, as previously reported [9,10], and formed three subgroups correlating with the year of isolation but not geographic location or poultry host.

Except for two isolates, the HAs of the Egyptian H5N8 viruses had PLREKRRKR/GLF at the HA cleavage site. A previous study of Egyptian viruses with this signature showed that they were HPAI with Intravenous Pathogenicity Indexes (IVPIs) ranging from 2.68 to 2.9 [51]. In contrast to a recently published study [48], no changes in glycosylation sites were recorded among the Egyptian H5N8 strains in this study.

Accumulation of random mutations and reassortment of gene segments are the main mechanisms employed for the continuous emergence of new variants and genotypes of avian influenza viruses. The Egyptian H5N8 viruses maintained many molecular signatures associated with avian adapted viruses, although some mammalian adaptation and virulence mutations were seen. In most isolates, the PDZ motif was absent, the PB1-F2 was truncated, and two mammalian virulence mutations NS1 (189N) and NS2 (31I), previously identified in Egyptian H5 viruses, were detected. Other mammalian adaptations and virulence features detected in Egyptian H5N8 were PB2 (504V), PB1 (13P), PA (127V and 672L), NP (398Q), M2 (64S), and NS1 (42S). Despite these signatures, no evidence for human infection with these viruses was detected [52].

Six different reassortant H5N8 viruses were detected in Egypt with evidence for multiple introductions. Only G4 became dominant in Egypt and was detected in the Delta and the upper part of Egypt in different production sectors from 2018 to 2021. Among six different genotypes, only G1 and G6 were previously identified in wild migratory birds in Egypt at the end of 2016 and the start of 2017. The relationship between the H5N8 viruses in domestic poultry in Egypt and the detected viruses in Egypt and European and Asian countries emphasizes the essential role of migratory birds in viral dissemination through the Black Sea–Mediterranean and East Asia–West Africa flyways. Interestingly, the 2020 virus of genotype G6 was closely similar to the H5N8 virus characterized in wild birds in Egypt in 2016, suggesting the maintenance of some genotypes across years. It is unclear whether this virus was maintained in Egypt or it was introduced into the country on two separate occasions. Despite the presence of this genotype across the years, we saw evidence of annual genotype displacement. Genotype replacement might be associated with the virulence tradeoff hypothesis, which indicates that lower virulence is associated with a potentially prolonged stage. Further studies are required to determine the significant effect of genetic reassortment among different genetic constellation forms of H5N8 viruses detected through virological features and virus replication characteristics in cultured cells (in vitro) and in different experimental animal models (in vivo). Such studies could help understand the dynamics of virus replacement.

Although the HAs of the Egyptian H5N8 viruses were genetically similar to those of the World Health Organization (WHO) 2.3.4.4b Candidate Vaccine Virus (CVV) A/Fujian-Sanyuan/21099/2017 (H5N6), they did not react well with the serum raised against it. Additional studies analyzing selected candidate vaccines against circulating strains as well as regular updating will be important for optimal public health and pandemic preparedness. Accordingly, the development of a new 2.3.4.4b CVV similar to A/Astrakhan/3212/2020 (H5N8) has been proposed by the WHO. The antigenic similarity of this virus and the Egyptian H5N6 viruses has not yet been determined.

As we previously predicted [53], several antigenically drifted H5N8 HPAIVs were isolated from flocks of chickens vaccinated with mismatched vaccine strains. This result supports the notion that vaccination can contribute to the selection of antigenically drifted strains. It is important to note that if vaccination against H5N8 viruses is adopted, vaccine candidates from local strains should be used to reduce the risk of emergence of vaccine escape mutants. This study also reiterates the need for enhanced surveillance and monitoring of avian influenza viruses in Egypt, given the requirement to evaluate the efficacy of currently used vaccines against newly introduced strains. Genetic and antigenic characterization of circulating AIVs in wild and domestic poultry is vital to predict and fight potential pandemics that could threaten public health.

## Figures and Tables

**Table 1:** Analysis of virulence determinants in the viral PB2, PB1, PA, NP, M2, NS1, and NS2 proteins.

Protein	aa Site	Virulent	Avirulent	Percentage	References
**PB2**	627	K	E	E (100%)	[23]
	147	L	M	I (100%)	[23]
	250	G	V	V (100%)	[23]
	504	V	I	V (100%)	[18]
	701	N	D	D (100%)	[24]
	591	K	Q	Q (100%)	[25]
**PB1**	317	I	M/V	M (100%)	[17,26]
**PA**	127	V	I	V (96.88%), I (3.12%)	[21]
	672	L	F	L (100%)	[19]
	100	R	V	V (100%)	[27]
	550	L	I	L (100%)	[18]
**NP**	470	R	K	K (100%)	[28]
**M2**	64	S/A/F	P	S (100%)	[21]
	69	P	L	P (100%)	[21]
**NS1**	42	S	A/P	S (100%)	[20]
	92	E	D	D (100%)	[26]
	103	L	F	F (97.9%), L (2.1%)	[29]
	106	I	M	M (100%)	[29]
	189	N	D/G	D (95.84%), N (4.16%)	[30]
	PDZ motif (227–230)	Presence	Deletion	Presence (22.9%), Deletion (77.1%)	
**NS2**	31	I	M	M (98%), (2%) I	[30]
	56	Y	H/L	H (100%)	[30]

**Table 2:** Analysis of genetic determinants of host range in the PB2, PB1, PA, NP, M1, M2, NS1, and NS2 proteins in H5N8 viruses. The host preference markers are shown.

Viral Protein	aa Site	Avian Preference	Mammalian Preference	Egyptian Strains	References
**PB2**	44	A	S	A (100%)	[17,31]
	64	M	T	M (100%)	[32]
	81	T	M	T (100%)	[31]
	199	A	S	A (100%)	[17,31]
	591	Q	K	Q (100%)	[25]
	627	E	K	E (100%)	[23]
	661	A	T	A (92.7%), V (7.3%)	[33]
	701	D	N	D (100%)	[24]
	702	K	R	K (100%)	[33]
**PB1**	13	L	P	P (100%)	[34]
	336	V	I	V (100%)	[17]
	375	N	S	N (100%)	[35]
**PA**	28	P	L	P (100%)	[36]
	55	D	N	D (100%)	[17,31]
	57	R	Q	R (100%)	[17]
	100	V	A	V (100%)	[37]
	133	E	G	E (100%)	[38]
	225	S	C	S (100%)	[39]
	241	C	Y	C (100%)	[40]
	268	L	I	L (100%)	[39]
	356	K	R	K (100%)	[17]
	382	E	D	E (100%)	[31,35]
	404	A	S	A (100%)	[17]
	409	S	N	S (100%)	[17,31]
	552	T	S	T (100%)	[39]
	615	K	L	K (29.2%), R (70.8%)	[41]
**HA**	222	Q	L	Q (100%)	[42]
	224	G	S	G (100%)	[42]
**NP**	33	V	I	V (94%), I (5%), D (1%)	[17,31]
	61	I	L	I (100%)	[31,39]
	109	I	V	I (100%)	[17]
	136	L	M	L (100%)	[31]
	214	R	K	R (100%)	[17,31]
	313	F	Y	F (100%)	[17,31]
	357	Q	K	Q (100%)	[17]
	372	E	D	E (100%)	[17]
	398	K	Q	Q (100%)	[17]
	455	D	E	D (94.8%), N (3.1%), E (2.1%)	[17]
**M1**	15	V	I	V (100%)	[43]
	115	V	I	V (89.6%), I (9.4%), T (1%)	[39]
	121	T	A	T (100%)	[39]
	137	T	A	T (100%)	[31,39]
**M2**	11	T	I	T (100%)	[17]
	16	E	G/D	E (94.8%), G (5.2%)	[31]
	20	S	N	S (100%)	[17,31]
	28	I	I/V	I (100%)	[31]
	57	Y	H	Y (100%)	[17]
	55	L	F	L (100%)	[44]
	86	V	A	V (100%)	[17]
**NS1**	227	E	K/R	G (22.9%), deletion (77.1%)	[45]
	full length	217	230	230 (22.9%)	

## Data Availability

The data presented in this study are available in the manuscript and supplementary material. All sequences presented in this study are openly available in GenBank under the accession numbers listed in Table S2.

## Supplementary Materials

The following supporting information can be downloaded at: https://www.mdpi.com/article/10.3390/microorganisms10030595/s1, Table S1: Summary of the epidemiological data of the Egyptian AI H5N8 viruses used in this study; Table S2: Summary of the genotyping of Egyptian AIV H5N8 genes analyzed in the current study with their accession numbers in GenBank; Table S3: Hemagglutination inhibition assay titers of polyclonal antibodies against different Egyptian H5N8 isolates.
