# Supplementary material for: Genetic and Antigenic Characteristics of Highly Pathogenic Avian Influenza A(H5N8) Viruses Circulating in Domestic Poultry in Egypt, 2017–2021
Source: Microorganisms. 2022 Mar 9;10(3):595. doi: 10.3390/microorganisms10030595 (PMC8948635; doi:10.3390/microorganisms10030595)
Supplement: Supplementary file 1 [file microorganisms-10-00595-s001.zip › microorganisms-1622858-supplementary.pdf]

Table S1. Summary of the epidemiological data of the Egyptian AI H5N8 viruses used in this study

H5N8 isolate	Governorate	Host	Health status	Sampling site	Date	Vaccination status
**A/duck/Egypt/F13666A/2017**	Fayoum	Duck	Healthy	Farm, free-range	1/26/2017	Unknown
**A/chicken/Egypt/N13717E/2017**	Menia	Chicken	Healthy	Farm, free-range	2/14/2017	Unknown
**A/chicken/Egypt/N13718C/2017**	Menia	Chicken	Healthy	Farm, free-range	2/14/2017	Unknown
**A/chicken/Egypt/N13720E/2017**	Menia	Chicken	Healthy	Farm, free-range	2/14/2017	Unknown
**A/chicken/Egypt/N13722/2017**	Menia	Chicken	Healthy	Live bird market	2/14/2017	Unknown
**A/chicken/Egypt/N13726/2017**	Menia	Chicken	Healthy	Live bird market	2/14/2017	Unknown
**A/chicken/Egypt/N13731D/2017**	Menia	Chicken	Healthy	Farm, free-range	2/14/2017	Unknown
**A/chicken/Egypt/N13731A/2017**	Menia	Chicken	Healthy	Farm, free-range	2/14/2017	Unknown
**A/chicken/Egypt/N13731C/2017**	Menia	Chicken	Healthy	Farm, free-range	2/14/2017	Unknown
**A/duck/Egypt/N13735A/2017**	Menia	Duck	Healthy	Farm, free-range	2/15/2017	Unknown
**A/duck/Egypt/N13736A/2017**	Menia	Duck	Healthy	Farm, free-range	2/15/2017	Unknown
**A/duck/Egypt/N13736E/2017**	Menia	Duck	Healthy	Farm, free-range	2/15/2017	Unknown
**A/chicken/Egypt/A13773E/2017**	Assiut	Chicken	Healthy	Farm, indoor	2/25/2017	Unknown
**A/chicken/Egypt/A13775C/2017**	Assiut	Chicken	Healthy	Farm, indoor	2/25/2017	Unknown
**A/goose/Egypt/A13779B/2017**	Assiut	Goose	Healthy	Farm, free-range	2/25/2017	Unknown
**A/duck/Egypt/H13779E/2017**	Assiut	Goose	Healthy	Farm, free-range	2/25/2017	Unknown
**A/chicken/Egypt/H13794D/2017**	Sohag	Chicken	Healthy	Farm, indoor	2/26/2017	Unknown
**A/chicken/Egypt/H13795A/2017**	Sohag	Chicken	Healthy	Farm, indoor	2/26/2017	Unknown
**A/chicken/Egypt/Q13804A/2017**	Kalyobiya	Chicken	Healthy	Farm, indoor	1/3/2017	H9N2&H5N1
**A/chicken/Egypt/F13829A/2017**	Fayoum	Chicken	Healthy	Farm, indoor	3/7/2017	Unknown
**A/chicken/Egypt/F13829B/2017**	Fayoum	Chicken	Healthy	Farm, indoor	3/7/2017	Unknown
**A/chicken/Egypt/M13844B/2017**	Monofiya	Chicken	Healthy	Farm, indoor	3/15/2017	H5N1
**A/chicken/Egypt/Q13845A/2017**	Kalyobiya	Chicken	Healthy	Farm, indoor	3/15/2017	H5N1
**A/chicken/Egypt/Q13936B/2017**	Kalyobiya	Chicken	Healthy	Farm, indoor	4/12/2017	H9N2&H5N1
**A/chicken/Egypt/Q13936C/2017**	Kalyobiya	Chicken	Healthy	Farm, indoor	4/12/2017	H9N2&H5N1
**A/chicken/Egypt/M14081D/2017**	Monofiya	Chicken	Healthy	Farm, indoor	5/10/2017	H9N2&H5N1
**A/chicken/Egypt/F14110D/2017**	Fayoum	Chicken	Healthy	Farm, indoor	5/16/2017	H9N2
**A/duck/Egypt/N14200A/2017**	Menia	Duck	Healthy	Farm, free-range	6/9/2017	Unknown
**A/duck/Egypt/N14205C/2017**	Menia	Duck	Healthy	Farm, indoor	6/9/2017	Unknown
**A/chicken/Egypt/A15037/2018**	Assiut	Chicken	Healthy	Live bird market	1/15/2018	Unknown
**A/chicken/Egypt/A15044/2018**	Assiut	Chicken	Healthy	Live bird market	1/15/2018	Unknown
**A/pigeon/Egypt/A15052/2018**	Assiut	Pigeon	Healthy	Live bird market	1/15/2018	Unknown
**A/duck/Egypt/F15089/2018**	Fayoum	Duck	Healthy	Live bird market	1/14/2018	Unknown
**A/duck/Egypt/F15092/2018**	Fayoum	Duck	Healthy	Live bird market	1/14/2018	Unknown
**A/chicken/Egypt/F15099/2018**	Fayoum	Chicken	Healthy	Live bird market	1/14/2018	Unknown
**A/chicken/Egypt/F15100/2018**	Fayoum	Chicken	Healthy	Live bird market	1/14/2018	Unknown
**A/chicken/Egypt/N15168C/2018**	Menia	Chicken	Dead	Farm, free-range	2/5/2018	Unknown
**A/chicken/Egypt/N15169A/2018**	Menia	Chicken	Dead	Farm, free-range	2/5/2018	Unknown
**A/chicken/Egypt/N15172D/2018**	Menia	Chicken	Dead	Farm, free-range	2/5/2018	Unknown
**A/chicken/Egypt/N15173A/2018**	Menia	Chicken	Dead	Farm, free-range	2/5/2018	Unknown
**A/chicken/Egypt/N15173B/2018**	Menia	Chicken	Dead	Farm, free-range	2/5/2018	Unknown
**A/chicken/Egypt/N15173C/2018**	Menia	Chicken	Dead	Farm, free-range	2/5/2018	Unknown

**A/chicken/Egypt/N15173D/2018**	Menia	Chicken	Dead	Farm, free-range	2/5/2018	Unknown
**A/chicken/Egypt/N15174A/2018**	Menia	Chicken	Dead	Farm, free-range	2/5/2018	Unknown
**A/chicken/Egypt/N15174B/2018**	Menia	Chicken	Dead	Farm, free-range	2/5/2018	Unknown
**A/chicken/Egypt/N15174C/2018**	Menia	Chicken	Dead	Farm, free-range	2/5/2018	Unknown
**A/chicken/Egypt/N15175C/2018**	Menia	Chicken	Dead	Farm, free-range	2/5/2018	Unknown
**A/chicken/Egypt/N15175A/2018**	Menia	Chicken	Dead	Farm, free-range	2/5/2018	Unknown
**A/chicken/Egypt/N15175B/2018**	Menia	Chicken	Dead	Farm, free-range	2/5/2018	Unknown
**A/chicken/Egypt/N15175D/2018**	Menia	Chicken	Dead	Farm, free-range	2/5/2018	Unknown
**A/chicken/Egypt/N15176A/2018**	Menia	Chicken	Dead	Farm, free-range	2/5/2018	Unknown
**A/chicken/Egypt/N15176B/2018**	Menia	Chicken	Dead	Farm, free-range	2/5/2018	Unknown
**A/chicken/Egypt/N15177A/2018**	Menia	Chicken	Dead	Farm, free-range	2/5/2018	Unknown
**A/chicken/Egypt/N15177D/2018**	Menia	Chicken	Dead	Farm, free-range	2/5/2018	Unknown
**A/chicken/Egypt/N15177C/2018**	Menia	Chicken	Dead	Farm, free-range	2/5/2018	Unknown
**A/chicken/Egypt/N15178B/2018**	Menia	Chicken	Dead	Farm, free-range	2/5/2018	Unknown
**A/chicken/Egypt/N15178A/2018**	Menia	Chicken	Dead	Farm, free-range	2/5/2018	Unknown
**A/chicken/Egypt/N15178C/2018**	Menia	Chicken	Dead	Farm, free-range	2/5/2018	Unknown
**A/chicken/Egypt/N15178D/2018**	Menia	Chicken	Dead	Farm, free-range	2/5/2018	Unknown
**A/chicken/Egypt/F15366B/2018**	Fayoum	Chicken	Dead	Farm, indoor	3/20/2018	H9N2
**A/duck/Egypt/A16368/2018**	Assiut	Duck	Healthy	Live bird market	11/24/2018	Unknown
**A/duck/Egypt/A16372/2018**	Assiut	Duck	Healthy	Live bird market	11/24/2018	Unknown
**A/chicken/Egypt/Q16684C/2019**	Kalyobiya	Chicken	Dead	Farm, indoor	1/30/2019	H9N2&H5N1
**A/chicken/Egypt/Q16710A/2019**	Kalyobiya	Chicken	Dead	Farm, indoor	2/13/2019	H9N2&H5N1
**A/chicken/Egypt/Q16710B/2019**	Kalyobiya	Chicken	Dead	Farm, indoor	2/13/2019	H9N2&H5N1
**A/chicken/Egypt/Q16710C/2019**	Kalyobiya	Chicken	Dead	Farm, indoor	2/13/2019	H9N2&H5N1
**A/chicken/Egypt/Q16711A/2019**	Kalyobiya	Chicken	Dead	Farm, indoor	2/13/2019	H9N2&H5N1
**A/chicken/Egypt/Q16711B/2019**	Kalyobiya	Chicken	Dead	Farm, indoor	2/13/2019	H9N2&H5N1
**A/chicken/Egypt/Q16711C/2019**	Kalyobiya	Chicken	Dead	Farm, indoor	2/13/2019	H9N2&H5N1
**A/chicken/Egypt/Q16712A/2019**	Kalyobiya	Chicken	Dead	Farm, indoor	2/13/2019	H9N2&H5N1
**A/duck/Egypt/Q16716A/2019**	Kalyobiya	Duck	Dead	Farm, indoor	2/13/2019	H9N2&H5N1
**A/duck/Egypt/N16717/2019**	Menia	Duck	Healthy	Live bird market	2/21/2019	Unknown
**A/duck/Egypt/N16719/2019**	Menia	Duck	Healthy	Live bird market	2/21/2019	Unknown
**A/duck/Egypt/N16720/2019**	Menia	Duck	Healthy	Live bird market	2/21/2019	Unknown
**A/duck/Egypt/N16721/2019**	Menia	Duck	Healthy	Live bird market	2/21/2019	Unknown
**A/duck/Egypt/N16722/2019**	Menia	Duck	Healthy	Live bird market	2/21/2019	Unknown
**A/chicken/Egypt/N16730/2019**	Menia	Chicken	Healthy	Live bird market	2/21/2019	Unknown
**A/chicken/Egypt/N16732/2019**	Menia	Chicken	Healthy	Live bird market	2/21/2019	Unknown
**A/duck/Egypt/A16793/2019**	Assiut	Duck	Healthy	Live bird market	2/27/2019	Unknown
**A/pigeon/Egypt/A16800/2019**	Assiut	Pigeon	Healthy	Live bird market	2/27/2019	Unknown
**A/pigeon/Egypt/A16804/2019**	Assiut	Pigeon	Healthy	Live bird market	2/27/2019	Unknown
**A/pigeon/Egypt/A16805/2019**	Assiut	Pigeon	Healthy	Live bird market	2/27/2019	Unknown
**A/chicken/Egypt/Q16807B/2019**	Kalyobiya	Chicken	Dead	Farm, indoor	3/2/2019	H9N2&H5N1
**A/chicken/Egypt/Q16807C/2019**	Kalyobiya	Chicken	Dead	Farm, indoor	3/2/2019	H9N2&H5N1
**A/chicken/Egypt/Q16807E/2019**	Kalyobiya	Chicken	Dead	Farm, indoor	3/2/2019	H9N2&H5N1
**A/chicken/Egypt/F17229A/2019**	Fayoum	Chicken	Dead	Farm, indoor	6/25/2019	H5N1
**A/chicken/Egypt/F17229B/2019**	Fayoum	Chicken	Dead	Farm, indoor	6/25/2019	H5N1
**A/chicken/Egypt/F17230A/2019**	Fayoum	Chicken	Dead	Farm, indoor	6/25/2019	H5N1

**A/chicken/Egypt/F17230B/2019**	Fayoum	Chicken	Dead	Farm, indoor	6/25/2019	H5N1
**A/chicken/Egypt/F17230C/2019**	Fayoum	Chicken	Dead	Farm, indoor	6/25/2019	H5N1
**A/chicken/Egypt/F17230D/2019**	Fayoum	Chicken	Dead	Farm, indoor	6/25/2019	H5N1
**A/chicken/Egypt/S18182C/2020**	Sharqeia	Chicken	Healthy	Farm, indoor	4/1/2020	Unknown
**A/Duck/Egypt/A19643/2021**	Assiut	Duck	Healthy	Live bird market	1/26/2021	Unknown
**A/chicken/Egypt/A19670/2021**	Assiut	Chicken	Healthy	Live bird market	2/10/2021	Unknown
**A/chicken/Egypt/A19671/2021**	Assiut	Chicken	Healthy	Live bird market	2/10/2021	Unknown
**A/chicken/Egypt/A19673/2021**	Assiut	Chicken	Healthy	Live bird market	2/10/2021	Unknown

Table S2. Summary of the genotyping of Egyptian AIV H5N8 genes analyzed in the current study with their accession numbers in GenBank.

H5N8 isolate	Genotype	PB2	PB1	PA	HA	NP	NA	M	NS
**A/duck/Egypt/F13666A/2017**	**G1**	MH498566	MH498601	MH498580	MH498622	MH498619	MH498574	MH498609	MH498564
**A/chicken/Egypt/N13717E/2017**	**G1**	OM333407	OM333406	OM333405	MH998454	OM333403	MH998455	OM333402	OM333404
**A/chicken/Egypt/N13718C/2017**	**G1**	OM333429	OM333428	OM333427	MH998450	OM333425	MH998451	OM333424	OM333426
**A/chicken/Egypt/N13720E/2017**	**G1**	OM333379	OM333378	OM333377	OM333372	OM333375	OM333374	OM333373	OM333376
**A/chicken/Egypt/N13722/2017**	**G1**	OM333323	OM333322	OM333321	MH998397	OM333319	MH998398	OM333318	OM333320
**A/chicken/Egypt/N13726/2017**	**G1**	OM333352	OM333351	OM333350	MH998391	OM333348	MH998392	OM333347	OM333349
**A/chicken/Egypt/N13731D/2017**	**G1**	OM333269	OM333268	OM333267	OM333262	OM333265	OM333264	OM333263	OM333266
**A/chicken/Egypt/N13731A/2017**	**G1**	OM333365	OM333364	OM333363	MH998395	OM333361	MH998396	OM333360	OM333362
**A/chicken/Egypt/N13731C/2017**	**G1**	OM333442	OM333441	OM333440	OM333435	OM333438	OM333437	OM333436	OM333439
**A/duck/Egypt/N13735A/2017**	**G1**	OM333393	OM333392	OM333391	MH998522	OM333389	MH998523	OM333388	OM333390
**A/duck/Egypt/N13736A/2017**	**G1**	OM333286	OM333285	OM333284	OM333279	OM333282	OM333281	OM333280	OM333283
**A/duck/Egypt/N13736E/2017**	**G1**	MH498579	MH498560	MH498616	MH498625	MH498576	MH498591	MH498583	MH498555
**A/chicken/Egypt/A13773E/2017**	**G1**	OM333346	OM333345	OM333344	OM333340	OM333343	OM333342	OM333341	OM333311
**A/chicken/Egypt/A13775C/2017**	**G1**	OM333423	OM333422	OM333421	MH998456	OM333419	MH998457	OM333418	OM333420
**A/goose/Egypt/A13779B/2017**	**G1**	OM333255	OM333254	OM333253	MH998508	OM333251	MH998509	OM333250	OM333252
**A/duck/Egypt/H13797E/2017**	**G1**	OM333278	OM333277	OM333276	MH998466	OM333274	MH998467	OM333273	OM333275
**A/chicken/Egypt/H13794D/2017**	**G1**	OM333359	OM333358	OM333357	OM333353	OM333356	OM333355	OM333354	OM333287
**A/chicken/Egypt/H13795A/2017**	**G1**	OM333294	OM333293	OM333292	MH998387	OM333291	MH998388	OM333290	OM333249
**A/chicken/Egypt/Q13804A/2017**	**G1**	MH498557	MH498582	MH498559	MH498577	MH498603	MH498607	MH498592	MH498628
**A/chicken/Egypt/F13829A/2017**	**G1**	OM333261	OM333260	OM333259	MH998500	OM333257	MH998501	OM333256	OM333258
**A/chicken/Egypt/F13829B/2017**	**G1**	OM350193	OM350192	OM350191	OM350186	OM350189	OM350188	OM350187	OM350190
**A/chicken/Egypt/M13844B/2017**	**G1**	OM333434	OM333433	OM333432	MH998464	OM333431	MH998465	OM333430	OM333416
**A/chicken/Egypt/Q13845A/2017**	**G1**	OM333302	OM333301	OM333300	OM333295	OM333298	OM333297	OM333296	OM333299
**A/chicken/Egypt/Q13936B/2017**	**G2**	OM333310	OM333309	OM333308	MH998506	OM333306	MH998507	OM333305	OM333307
**A/chicken/Egypt/Q13936C/2017**	**G2**	OM333339	OM333338	OM333337	OM333333	OM333336	OM333335	OM333334	OM333366
**A/chicken/Egypt/M14081D/2017**	**G3**	OM333317	OM333316	OM333315	MH998462	OM333313	MH998463	OM333312	OM333314
**A/chicken/Egypt/F14110D/2017**	**G3**	OM333415	OM333414	OM333413	OM333408	OM333411	OM333410	OM333409	OM333412
**A/duck/Egypt/N14200A/2017**	**G1**	OM333401	OM333400	OM333399	OM333394	OM333397	OM333396	OM333395	OM333398
**A/duck/Egypt/N14205C/2017**	**G3**	OM333331	OM333330	OM333329	OM333324	OM333327	OM333326	OM333325	OM333328
**A/chicken/Egypt/A15037/2018**	**G3**	OL354971	OL354972	OL354973	OL354974	OL354975	OL354976	OL354977	OL354978
**A/chicken/Egypt/A15044/2018**	**G3**	OL354939	OL354940	OL354941	OL354942	OL354943	OL354944	OL354945	OL354946
**A/pigeon/Egypt/A15052/2018**	**G3**	OL354947	OL354948	OL354949	MN038184	OL354950	OL354951	OL354952	OL354953
**A/duck/Egypt/F15089/2018**	**G3**	OL354885	OL354886	OL354887	OL354888	OL354889	OL354890	OL354891	OL354892
**A/duck/Egypt/F15092/2018**	**G3**	OL354914	OL354915	OL354916	OL354917	OL354918	OL354919	OL354920	OL354921

**A/chicken/Egypt/F15099/2018**	**G3**	OL354930	OL354931	OL354932	MN038194	OL354933	OL354934	OL354935	OL354936
**A/chicken/Egypt/F15100/2018**	**G3**	OL354547	OL354548	OL354549	OL354550	OL354551	OL354552	OL354553	OL354554
**A/chicken/Egypt/N15168C/2018**	**G3**	OL354523	OL354524	OL354525	OL354526	OL354527	OL354528	OL354529	OL354530
**A/chicken/Egypt/N15169A/2018**	**G3**	OL354730	OL354731	OL354732	OL354733	OL354734	OL354735	OL354736	OL354737
**A/chicken/Egypt/N15172D/2018**	**G3**	OM333387	OM333386	OM333385	OM333380	OM333383	OM333382	OM333381	OM333384
**A/chicken/Egypt/N15173A/2018**	**G3**	OL354477	OL354478	OL354479	OL354480	OL354481	OL354482	OL354483	OL354484
**A/chicken/Egypt/N15173B/2018**	**G3**	OL354563	OL354564	OL354565	OL354566	OL354567	OL354568	OL354569	OL354570
**A/chicken/Egypt/N15173C/2018**	**G3**	OL354555	OL354556	OL354557	OL354558	OL354559	OL354560	OL354561	OL354562
**A/chicken/Egypt/N15173D/2018**	**G3**	OL354500	OL354501	OL354502	MN038195	OL354503	OL354504	OL354505	OL354506
**A/chicken/Egypt/N15174A/2018**	**G3**	OL354539	OL354540	OL354541	OL354542	OL354543	OL354544	OL354545	OL354546
**A/chicken/Egypt/N15174B/2018**	**G3**	OL354831	OL354832	OL354833	OL354834	OL354835	OL354836	OL354837	OL354838
**A/chicken/Egypt/N15174C/2018**	**G3**	OL354446	OL354447	OL354448	OL354449	OL354450	OL354451	OL354452	OL354453
**A/chicken/Egypt/N15175C/2018**	**G3**	OL354454	OL354455	OL354456	OL354457	OL354458	OL354459	OL354460	OL354461
**A/chicken/Egypt/N15175A/2018**	**G3**	OL354823	OL354824	OL354825	OL354826	OL354827	OL354828	OL354829	OL354830
**A/chicken/Egypt/N15175B/2018**	**G3**	OL354839	OL354840	OL354841	OL354842	OL354843	OL354844	OL354845	OL354846
**A/chicken/Egypt/N15175D/2018**	**G3**	OL354862	OL354863	OL354864	OL354865	OL354866	OL354867	OL354868	OL354869
**A/chicken/Egypt/N15176A/2018**	**G3**	OL354515	OL354516	OL354517	OL354518	OL354519	OL354520	OL354521	OL354522
**A/chicken/Egypt/N15176B/2018**	**G3**	OL354619	OL354620	OL354621	OL354622	OL354623	OL354624	OL354625	OL354626
**A/chicken/Egypt/N15177A/2018**	**G3**	OL354492	OL354493	OL354494	OL354495	OL354496	OL354497	OL354498	OL354499
**A/chicken/Egypt/N15177D/2018**	**G3**	OL354970	OL354770	OL354771	OL354772	OL354773	OL354774	OL354775	OL354776
**A/chicken/Egypt/N15177C/2018**	**G3**	OL354754	OL354755	OL354756	OL354757	OL354758	OL354759	OL354760	OL354761
**A/chicken/Egypt/N15178B/2018**	**G3**	OL354507	OL354508	OL354509	OL354510	OL354511	OL354512	OL354513	OL354514
**A/chicken/Egypt/N15178A/2018**	**G3**	OL354611	OL354612	OL354613	OL354614	OL354615	OL354616	OL354617	OL354618
**A/chicken/Egypt/N15178C/2018**	**G3**	OL354603	OL354604	OL354605	OL354606	OL354607	OL354608	OL354609	OL354610
**A/chicken/Egypt/N15178D/2018**	**G3**	OL354587	OL354588	OL354589	OL354590	OL354591	OL354592	OL354593	OL354594
**A/chicken/Egypt/F15366B/2018**	**G3**	OL354815	OL354816	OL354817	OL354818	OL354819	OL354820	OL354821	OL354822
**A/duck/Egypt/A16368/2018**	**G4**	OL354922	OL354923	OL354924	OL354925	OL354926	OL354927	OL354928	OL354929
**A/duck/Egypt/A16372/2018**	**G4**	OL354962	OL354963	OL354964	OL354965	OL354966	OL354967	OL354968	OL354969
**A/chicken/Egypt/Q16684C/2019**	**G3**	OM324349	OM324350	OM324351	OM324352	OM324353	OM324354	OM363258	OM324355
**A/chicken/Egypt/Q16710A/2019**	**G4**	OL366029	OL366030	OL366031	MW137743	MW137744	OM333332	MW137742	MW137745
**A/chicken/Egypt/Q16710B/2019**	**G4**	MW137657	OM333248	MW137663	MW137660	MW137662	MW137659	MW137661	MW137658
**A/chicken/Egypt/Q16710C/2019**	**G4**	MT261427	MT261429	MT261426	MT261424	MT261423	MT261425	MT261428	MT261422
**A/chicken/Egypt/Q16711A/2019**	**G4**	MT261431	MT261435	MT261434	MT261436	MT261432	MT261433	MT261437	MT261430
**A/chicken/Egypt/Q16711B/2019**	**G4**	MT261411	MT261410	MT261416	MT261415	MT261413	MT261414	MT261417	MT261412
**A/chicken/Egypt/Q16711C/2019**	**G4**	MT261503	MT261510	MT261508	MT261509	MT261506	MT261505	MT261507	MT261504
**A/chicken/Egypt/Q16712A/2019**	**G4**	MT261528	MT261527	MT261531	MT261534	MT261532	MT261533	MT261530	MT261529
**A/duck/Egypt/Q16716A/2019**	**G4**	MT261450	MT261452	MT261449	MT261451	MT261447	MT261446	MT261453	MT261448
**A/duck/Egypt/N16717/2019**	**G5**	MT261524	MT261522	MT261520	MT261526	MT261523	MT261521	MT261519	MT261525
**A/duck/Egypt/N16719/2019**	**G5**	MT261403	MT261406	MT261408	MT261405	MT261402	MT261409	MT261404	MT261407
**A/duck/Egypt/N16720/2019**	**G5**	MT261454	MT261460	MT261457	MT261458	MT261455	MT261459	MT261456	MT261461
**A/duck/Egypt/N16721/2019**	**G5**	MW137839	OM333417	MW137834	MW137837	MW137836	MW137838	MW137835	MW137833
**A/duck/Egypt/N16722/2019**	**G5**	MT261556	MT261557	MT261553	MT261558	MT261552	MT261555	MT261554	MT261551
**A/chicken/Egypt/N16730/2019**	**G4**	MT261474	MT261477	MT261478	MT261475	MT261471	MT261473	MT261472	MT261476
**A/chicken/Egypt/N16732/2019**	**G4**	MT261394	MT261398	MT261395	MT261396	MT261399	MT261400	MT261401	MT261397
**A/duck/Egypt/A16793/2019**	**G4**	MT261708	MT261707	MT261705	MT261710	MT261706	MT261711	MT261712	MT261709
**A/pigeon/Egypt/A16800/2019**	**G4**	MW137850	MW137847	MW137848	MW137849	OL366047	MW137851	OL366048	MW137846

**A/pigeon/Egypt/A16804/2019**	**G4**	MW137772	OL366027	MW137774	MW137775	OL366028	OM333303	OM333304	MW137773
**A/pigeon/Egypt/A16805/2019**	**G4**	MW137654	OL366049	MW137656	MW137652	OL366050	MW137653	OM333371	MW137655
**A/chicken/Egypt/Q16807B/2019**	**G4**	MW137685	MW137684	MW137682	MW137686	OM333247	MW137683	OL366051	MW137681
**A/chicken/Egypt/Q16807C/2019**	**G4**	MW137798	MW137797	MW137796	MW137792	MW137794	MW137795	OL366052	MW137793
**A/chicken/Egypt/Q16807E/2019**	**G4**	MT261580	MT261576	MT261577	MT261579	MT261578	MT261575	MT261581	MT261582
**A/chicken/Egypt/F17229A/2019**	**G4**	MW137843	MW137840	OL366040	MW137842	OL366041	OM333289	OM333288	MW137841
**A/chicken/Egypt/F17229B/2019**	**G4**	MW137855	OM333368	OM333367	MW137853	OM333369	OM333370	MW137852	MW137854
**A/chicken/Egypt/F17230A/2019**	**G4**	MW137934	MW137935	OL354531	MW137933	OL354937	MW137936	OL354938	MW137937
**A/chicken/Egypt/F17230B/2019**	**G4**	MW137804	MW137805	MW137799	MW137802	MW137801	MW137803	OL354440	MW137800
**A/chicken/Egypt/F17230C/2019**	**G4**	MW137887	MW137885	MW137886	MW137889	MW137883	MW137884	OL354877	MW137888
**A/chicken/Egypt/F17230D/2019**	**G4**	MW137815	MW137813	OL354627	MW137812	OL354900	OL354628	OL354901	MW137814
**A/chicken/Egypt/S18182C/2020**	**G6**	OL354571	OL354572	OL354573	OL354574	OL354575	OL354576	OL354577	OL354578
**A/duck/Egypt/A19643/2021**	**G4**	OL354979	OL354980	OL354981	OL354982	OL354983	OL354984	OL354985	OL354986
**A/chicken/Egypt/A19670/2021**	**G4**	OM363260	OL353695	OL353694	OL353696	OL353692	OL353697	OL353691	OL353693
**A/chicken/Egypt/A19671/2021**	**G4**	OL366044	OM363259	OL354905	OL366043	OL354903	OL366042	OL354902	OL354904
**A/chicken/Egypt/A19673/2021**	**G4**	OL354898	OL354897	OL354896	OL354899	OL354894	OL354439	OL354893	OL354895

Table S3. Hemagglutination inhibition assay titers of polyclonal antibodies against different Egyptian H5N8 isolates.

**Antigen names**	**F.2015-48-A/SICHUAN/26221/2014 (H5N6)**	**F.2015-7 -A/DK/ENGLAND/36254/2014 (H5N8)**	**F.2016-16 - A/GYRFALCON/WA/410886/2014 (H5N8)**	**F.2015-13 -A/CK/KUMAMOTO/1-7/14518356 (H5N8)**	**A/CHICKEN/VIETNAM/NCVD-15A59/2015 (H5N6)**	**F.2019-47- A/HUBEI/29578/2016 (H5N6)**	**F.2019-50- A/FUJIAN-SANYUAN/21099/2017 (H5N6)**	**G19931 A/SNOW GOOSE/MO/CC15-84A/2015 (H5N2)**
A/CHICKEN/EGYPT/A13773E/2017 (H5N8)	<10	80	80	160	<10	<10	<10	40
A/CHICKEN/EGYPT/F13829A/2017(H5N8)	10	80	80	160	10	<10	10	40
A/CHICKEN/EGYPT/H13794D/2017 (H5N8)	10	160	320	640	10	<10	20	80
A/CHICKEN/EGYPT/H13795A/2017 (H5N8)	<10	160	160	320	<10	<10	<10	40
A/CHICKEN/EGYPT/N13717E/2017(H5N8)	10	160	320	320	<10	<10	10	80
A/CHICKEN/EGYPT/N13720E/2017(H5N8)	<10	80	80	160	<10	<10	<10	40
A/CHICKEN/EGYPT/N13726/2017(H5N8)	10	80	160	320	10	<10	<10	40
A/CHICKEN/EGYPT/M13844B/2017(H5N8)	10	160	320	640	<10	<10	<10	80
A/CHICKEN/EGYPT/N13718C/2017(H5N8)	<10	160	160	320	<10	<10	10	80
A/CHICKEN/EGYPT/N13731A/2017(H5N8)	10	160	640	640	10	<10	20	80

A/CHICKEN/EGYPT/N13722/2017(H5N8)	<10	160	640	640	<10	<10	<10	40
A/CHICKEN/EGYPT/A15042/2018(H5N8)	20	160	160	320	<10	<10	20	80
A/CHICKEN/EGYPT/A15044/2018 (H5N8)	20	80	160	320	10	<10	<10	40
A/DUCK/EGYPT/F15089/2018 (H5N8)	<10	40	80	160	<10	<10	<10	40
A/CHICKEN/EGYPT/Q16710C/2019(H5N8)	<10	80	80	160	<10	<10	<10	40
A/CHICKEN/EGYPT/F14110D/2017(H5N8)	80	80	160	160	80	<10	40	40
A/DUCK/EGYPT/N14200A/2017(H5N8)	160	160	160	320	40	<10	40	80
A/DUCK/EGYPT/N14205C/2017(H5N8)	40	40	40	80	20	<10	<10	40
A/CHICKEN/EGYPT/F15099/2018 (H5N8)	10	40	10	80	<10	<10	<10	20
A/CHICKEN/EGYPT/N15173B/2018 (H5N8)	10	40	10	80	<10	<10	<10	40
A/CHICKEN/EGYPT/N15175C/2018 (H5N8)	<10	40	10	40	10	<10	<10	40
A/CHICKEN/EGYPT/N15176A/2018 (H5N8)	10	20	10	40	10	<10	<10	40
A/PIGEON/EGYPT/A15052/2018 (H5N8)	40	80	80	160	20	<10	10	40
A/DUCK/EGYPT/Q16716A/2019(H5N8)	<10	20	<10	20	40	<10	<10	40
A/PIGEON/EGYPT/A16800/2019(H5N8)	160	80	160	160	160	<10	80	40
A/PIGEON/EGYPT/A16805/2019(H5N8)	<10	40	<10	40	<10	<10	<10	20
A/CHICKEN/EGYPT/S18182C/2020 (H5N8)	10	20	20	40	<10	<10	<10	10
A/CHICKEN/EGYPT/A19670/2021/ (H5N8)	80	40	160	160	80	<10	40	80
A/CHICKEN/EGYPT/A19671/2021/ (H5N8)	160	80	160	320	80	<10	80	160
